# Metabolic consequences for mice lacking Endosialin: LC–MS/MS-based metabolic phenotyping of serum from C56Bl/6J Control and CD248 knock‐out mice

**DOI:** 10.1007/s11306-020-01764-1

**Published:** 2021-01-18

**Authors:** Emily G. Armitage, Alan Barnes, Kieran Patrick, Janak Bechar, Matthew J. Harrison, Gareth G. Lavery, G. Ed Rainger, Christopher D. Buckley, Neil J. Loftus, Ian D. Wilson, Amy J. Naylor

**Affiliations:** 1 Shimadzu Corporation, Manchester, UK; 2 Institute of Inflammation and Ageing, University of Birmingham, Birmingham, UK; 3 Institute of Cardiovascular Sciences, University of Birmingham, Birmingham, UK; 4 Department of Metabolism, Digestion and Reproduction, Imperial College, London, UK; 5 Kennedy Institute for Rheumatology, University of Oxford, Oxford, UK; 6 Institute of Metabolism and Systems Research, University of Birmingham, Birmingham, UK

**Keywords:** CD248, Endosialin, High fat diet, HRAM UHPLC–MS/MS

## Abstract

**Introduction:**

The Endosialin/CD248/TEM1 protein is expressed in adipose tissue and its expression increases with obesity. Recently, genetic deletion of CD248 has been shown to protect mice against atherosclerosis on a high fat diet.

**Objectives:**

We investigated the effect of high fat diet feeding on visceral fat pads and circulating lipid profiles in CD248 knockout mice compared to controls.

**Methods:**

From 10 weeks old, CD248^−/−^ and ^+/+^ mice were fed either chow (normal) diet or a high fat diet for 13 weeks. After 13 weeks the metabolic profiles and relative quantities of circulating lipid species were assessed using ultra high performance liquid chromatography-quadrupole time-of flight mass spectrometry (UHPLC–MS) with high resolution accurate mass (HRAM) capability.

**Results:**

We demonstrate a specific reduction in the size of the perirenal fat pad in CD248^−/−^ mice compared to CD248^+/+^, despite similar food intake. More strikingly, we identify significant, diet-dependent differences in the serum metabolic phenotypes of CD248 null compared to age and sex-matched wildtype control mice. Generalised protection from HFD-induced lipid accumulation was observed in CD248 null mice compared to wildtype, with particular reduction noted in the lysophosphatidylcholines, phosphatidylcholines, cholesterol and carnitine.

**Conclusions:**

Overall these results show a clear and protective metabolic consequence of CD248 deletion in mice, implicating CD248 in lipid metabolism or trafficking and opening new avenues for further investigation using anti-CD248 targeting agents.

**Supplementary information:**

The online version of this article (10.1007/s11306-020-01764-1) contains supplementary material, which is available to authorized users.

## Introduction

CD248 (Endosialin/Tumour Endothelial Marker 1) is a transmembrane glycoprotein expressed widely by stromal cells including pericytes (Tomkowicz et al. 2010; Naylor et al. 2014), fibroblasts (MacFadyen et al. 2005), osteoblasts (Naylor et al. 2012) and adipocytes (Petrus et al. 2019). CD248 expression is high on all mesenchymal cells in embryonic and new-born tissues but is expressed at very low levels postnatally in normal tissue (Rupp et al. 2006). This expression is markedly upregulated in cancer-associated fibroblasts (Nanda et al. 2006; Park et al. 2020), sites of active inflammation (Maia et al. 2011), healing (Hong et al. 2019) and during fibrosis of several organs including the liver and kidney (Smith et al. 2011; Wilhelm et al. 2016). The upregulation of CD248 at sites of tissue remodelling implies a role for this protein in the process.

Despite the discovery of CD248 several decades ago, its precise function and method of influence on the tissue remodelling process remains somewhat elusive. Investigation into its function in the regulation of vascular modelling and remodelling (Simonavicius et al. 2012; Naylor et al. 2014) has provided evidence to suggest that it exerts these effects through response to hypoxia, via the Hif1α-binding region on its promoter (Ohradanova et al. 2008) and that it has a role in transmission/potentiation of platelet-derived growth factor (PDGF) signalling via an unknown mechanism (Tomkowicz et al. 2010; Naylor et al. 2012, 2014; Benedetto et al. 2018).

Recently, a role for CD248 in adipocyte accumulation (Petrus et al. 2019) and atherosclerosis (Hasanov et al. 2017) has been identified. To determine whether CD248 was involved in the formation of atheroma, we previously crossed the CD248 knockout mouse with the ApoE^−/−^ model of atherosclerosis. After 13 weeks of high fat diet feeding, as expected, ApoE^−/−^ animals demonstrated marked formation of atheromatous lesions across the whole aorta. Importantly, there was significant reduction in plaque burden in the CD248/ApoE double knockout animals (Fig. S1), as has been reported previously (Hasanov et al. 2017). Interestingly, the descending aorta showed sensitivity to CD248 knockout, while disease in the arch was refractory to its loss. Analysis of plaque burden at the aortic root revealed that ApoE^−/−^ mice exhibited robust formation of plaques at this anatomical site, whereas disease was significantly reduced in ApoE^−/−^ CD248^−/−^ mice (Fig. S1). Once again, the mechanism by which CD248 exerts its effects is not clear, however it is clear that genetic deletion is protective in these settings, with CD248 knockout mice showing reduced atherosclerosis (Fig. S1 and Hasanov et al. 2017), reduced weight gain and diabetes (as measured by glucose tolerance tests) on a high fat diet (Petrus et al. 2019). Given this evidence that CD248 affects metabolism, we investigated the effect of high fat diet feeding on fat pad accumulation and blood lipid profiles using untargeted UHPLC–MS/MS, employing high resolution accurate mass (HRAM) to aid metabolite detection and analysed serum from age and sex-matched wildtype (C56Bl/6J) and CD248 null mice following 13 weeks chow or high fat diet (HFD) feeding.

## Materials and methods

### Chemicals and reagents

LC–MS grade methanol (MeOH), water, and additives (ammonium formate and formic acid) were purchased from Sigma Aldrich, Gillingham, UK.

### In vivo study design

All animal experiments were performed in accordance with U.K. laws [Animal (Scientific Procedures) Act 1986] and with the approval of the Local Ethics Committees at the University of Birmingham. Every effort has been made to present all data in accordance with the ARRIVE guidelines (NC3Rs).

The generation and genotyping of CD248^−/−^ mice has been described previously (Nanda et al. 2006). Mice were backcrossed for 10 generations onto a C57Bl/6J genetic background and were maintained on this background by regular refreshing of the colony with wildtype animals (Charles River, UK). SNP analysis (Transnetyx, Inc.) confirmed 91.6% C57Bl/6J alleles with the remaining percentage attributed to C57Bl/6N alleles. WT C57Bl/6J mice were purchased aged 4–6 weeks from Harlan, UK.

Mice were maintained on a 12 h light/dark cycle at 21 °C with ad libitum access to food and water. Mice were fed either a standard chow diet (10% kcal from fat), high in fibre containing complex carbohydrates, with fats from a variety of vegetable sources or a high fat diet (42% kcal from fat), consisting of amino acids supplemented with casein, corn starch, maltodextrose or sucrose, and soybean oil or lard. Chow diet: Special Diets Services U.K. RM3 (P) 801700. HFD: Special Diets Services U.K. Western RD (P) 829100, comprising: g% (w/w): sucrose 33.9%, milk fat 20%, casein 19.5%, maltodextrin 10%, corn starch 5%, cellulose 5%, corn oil 1%, calcium carbonate 0.4%, l-cystine 0.3%, choline bitartrate 0.2%, cholesterol 0.15%, antioxidant 0.01%, AIN-76-mx 3.5%, AIN-76A-VX 1%.

Male and female mice were assessed and the data from all mice (n = 49) used in the study are presented (no mice were excluded from the analysis): C57Bl/6J CD248^+/+^ wildtype male n = 14 (9 HFD + 5 chow), female n = 12 (6 HFD + 6 chow); C57Bl/6J CD248^−/−^ knockout male n = 11 (6 HFD + 5 chow), female n = 12 (6 HFD + 6 chow). All mice were fed a chow diet post-weaning until 10 weeks of age. At 10 weeks old mice were assigned randomly to experimental groups as cages and fed either a chow or high fat diet ad lib. for 13 weeks. Animals were housed in specific pathogen free conditions within individually ventilated cages (IVC) according to sex, genotype and diet, with each group containing between 4 and 6 mice. Food intake was assessed fortnightly by single housing mice for 24 h in metabolic cages. Water and food reservoirs were weighed (g) at the start and end of the 24 h period to calculate intake. Urine (mL) and faeces (g) collected during this period were also measured. Following 13 weeks of high fat diet feeding, 23 week-old mice were anaesthetised with isoflurane and a bolus serum blood sample obtained through cardiac puncture. Mice were sacrificed by cervical dislocation and fat pads (per-renal and gonadal) were removed and weighed.

### Sample preparation

Blood samples were allowed to clot for 1 h at room temperature and were then centrifuged at 3000 × g for 10 min in a refrigerated centrifuge (4 °C). The resulting serum supernatants were stored at − 80℃ prior to analysis. Samples (130 µL) were prepared for UHPLC–MS/MS analysis by mixing with 3 volumes of MeOH to precipitate proteins, followed by centrifugation at 16,000 × g for 15 min. The supernatants (450 µL) were then evaporated to dryness using a vacuum centrifuge. For aqueous metabolite extraction, 50 µL of water was added to each dried sample and samples were vortexed, followed by 10 min chilled sonication and centrifugation at 16,000 × g for 20 min. The aqueous fractions (45 µL supernatant) were collected into fresh Eppendorf tubes and the remaining pellets were reconstituted in 80 µL MeOH for organic metabolite extraction. Samples were vortexed followed by 10 min chilled sonication and centrifugation at 16,000 × g for 20 min. The organic fractions (75 µL supernatant) were added to the aqueous fractions and samples were vortexed, centrifuged at 16,000 × g for 20 min and supernatants (110 µL) collected into LC–MS vials for analysis. Aliquots (10 µL) of each sample were taken and combined into a pooled sample to be used for LC column conditioning and as a quality control (QC) sample to monitor the quality of the analysis (Sangster et al. 2006, Gika et al. 2007, Broadhurst et al. 2018) (see below).

### UHPLC–MS/MS

Metabolic profiling was performed using a Nexera (Prominence compatibility mode) LC system coupled to an LC–MS-IT-TOF mass spectrometer (Shimadzu Corporation, Kyoto, Japan) equipped with an electrospray ion source with positive and negative ion mode switching (100 ms switching time). Samples (0.5 µL) were injected in a randomised order starting with six injections of the pooled QC and a re-injection of the QC after every five sample injections. A Phenomenex Kinetex XB C_18_ column (2.1 × 100 mm; particle size 1.7 µm) was maintained at 50 °C. The chromatographic system used a binary solvent system delivered as a gradient of Solvent A (20 mM ammonium formate in water + 0.1% formic acid) and Solvent B (20 mM ammonium formate in MeOH + 0.1% formic acid) at a flow rate of 0.6 mL/min. The initial gradient conditions were 2% B for 1 min increasing to 40% B by 2 min followed by a pre-programmed non-linear concave gradient profile (setting − 3 in LabSolutions, see Fig. S3) to 92.55% B by 25 min. At 32 min the gradient was further increased to 95% B before the re-equilibration phase at 100% B by 33 min. The solvent composition was then held at 100% B for 19 min in order to wash the column and minimize the impact of late eluting compounds (particularly triacylglycerols) on subsequent analyses. At the end of this wash period the column was returned to 2% B over the next 3 min, making a total cycle time of 55 min per sample. Positive and negative ion mode mass spectra were acquired within a cycle time of 0.6 s over a mass range of m/z 70–1200. The ion source temperature was 250 °C, heated capillary temperature was 230 °C, electrospray voltage was 4.5 kV, electrospray nebulization gas flow was 1.5 L/min and detector voltage was 1.65 kV. An additional method was applied to acquire data dependent acquisition (DDA)-MS/MS data for the pooled QC sample to support metabolite annotation. The LC method was the same as described above except the injection volume was 2 µL. Data were acquired in positive ion mode with MS (m/z 70–2000) and MS/MS (m/z 50–1250) scans within a cycle time of 0.8 s.

### Data analysis

Profiling Solution software (Shimadzu Corporation, Kyoto, Japan) was used to analyse positive ion mode data from the untargeted UHPLC–MS analysis. Metabolic features (mass/retention time pairs) were detected above a threshold of 20,000 counts in the MS full scan data. The first 30 min of acquisition contained relevant metabolites and lipids that were reproducibly detected and this section of the data was considered from each sample. Detected features in all samples were aligned using a 20 mDa, 0.2 min tolerance to produce a peak area data matrix for statistical analysis. The data matrix was subsequently filtered using accepted and established criteria for QCs (Gika et al. 2007; Broadhurst et al. 2018) to consider only those features present in at least 50% of the QC samples with less than 30% RSD amongst them (calculated from QCs (n = 12) injected after every 5 biological samples not including the initial QCs used to prepare the system). Data were subsequently filtered to include only features in at least 75% of one of the four groups: CD248^+/+^ or CD248^−/−^ fed a chow or high fat diet.

Precision of the analytical data was visually inspected based on QC samples. Principal Components analysis (PCA) was performed using MetaboAnalyst software (https://www.metaboanalyst.ca) (Chong et al. 2019). Data were further processed with the QCs removed from the data matrix to assess the biological impact of chow and high fat diets on the CD248^+/+^ and CD248^−/−^ mice. PCA, heatmap and ANOVA analyses were performed to determine features of statistical significance. Significant features and biochemically related features were putatively identified using Metlin and LipidMaps structure database (LMSD) by comparison of the accurate mass and by relevant DDA-MS/MS spectra where available. All annotations satisfied the Metabolomics Standards Initiative (MSI) level 2 criteria, defined as putatively annotated compounds without chemical reference standards and based upon physicochemical properties and spectral similarity with public spectral libraries (Sumner et al. 2007; Spicer et al. 2017). All annotations are summarised in Supplementary Table 1 and an example of an annotated HRAM chromatogram is shown in Fig. S3.

## Results and discussion

### CD248 knockout mice show reduced weight gain and adiposity on HFD

**Fig. 1:**
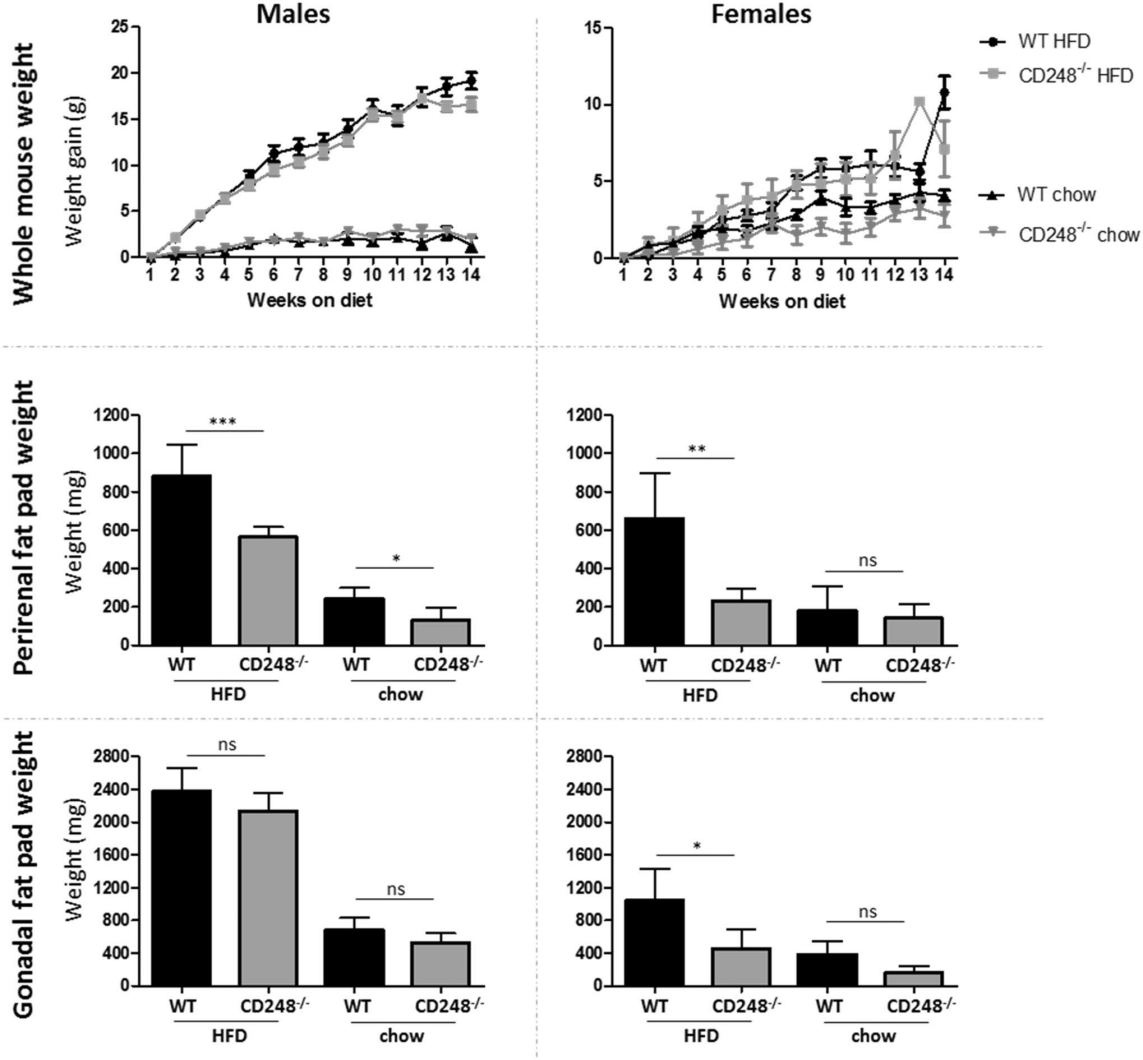
Male (left) and female (right) wildtype (WT, CD248^+/+^) and CD248^−/−^ mice aged 10 weeks old were fed high fat diet (HFD) or chow for 13 weeks. Weekly weight gain from baseline (top graphs) and final wet weights of two peri-visceral fat pads: perirenal/retroperitoneal fat pad (middle graphs) and the gonadal/epididymal fat pads (bottom graphs). Significance was measured by t-test: *p < 0.05; **p < 0.01; ***p < 0.001; *ns* non-significant

**Fig. 2:**
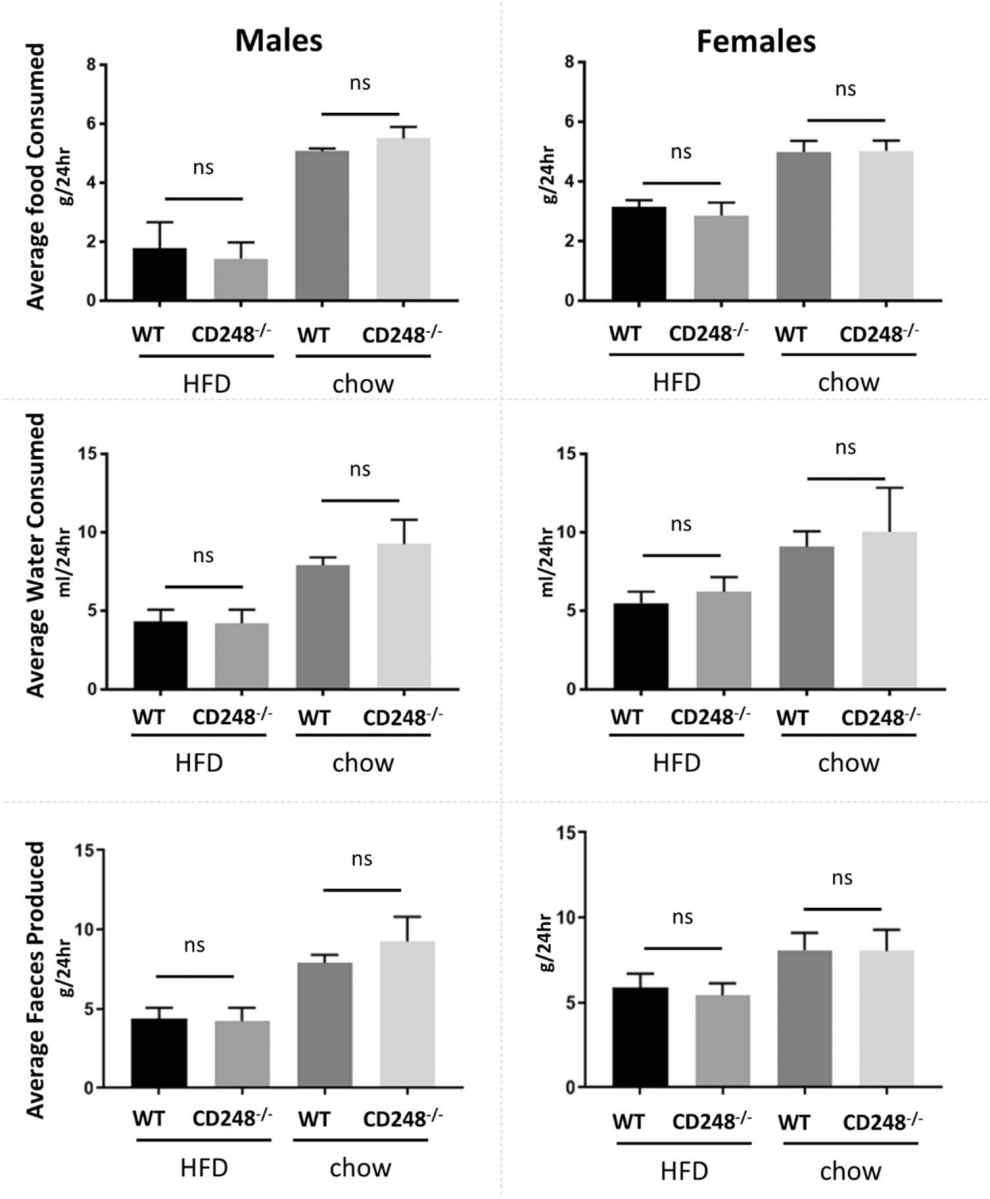
Food and water intake. Male (left) and female (right) wildtype (WT, CD248^+/+^) and CD248^−/−^ mice aged 10 weeks old were fed either high fat diet (HFD) or chow for 13 weeks. Every 2 weeks, 24 h-consumption of food (top graphs) and water (middle graphs), and quantity of faeces produced (lower graphs), were assessed in metabolic cages. Data shown is the mean ± standard deviation of all of the 5 time-points assessed. Significance was measured by t-test: *ns* non-significant

As part of ongoing research into the function of the CD248 gene on the *in vivo* metabolism of fat, we undertook a study to investigate the effects of a HFD on mice where this gene had been knocked out compared to control mice. Petrus et al. (2019) have previously reported that the CD248^−/−^ mouse has reduced weight gain on a HFD. Our data are broadly supportive of this phenotype. Although we did not see a statistically significant difference in whole mouse weights, there was a clear reduction in fat accumulation in the peri-visceral fat pads. This phenotype was evident in both males and females (Fig. 1). To ensure that this difference in adipose accumulation was not caused by differential food intake between the genotypes, mice were individually assessed (by singly housing in metabolic cages for 48 h), fortnightly for consumption of diet and water and for quantity of faeces produced. No significant effect of genotype was seen in any of these parameters for either male or female mice (Fig. 2).

### Dietary composition has differential effects on serum lipids in CD248^−/−^ mice

**Fig. 3:**
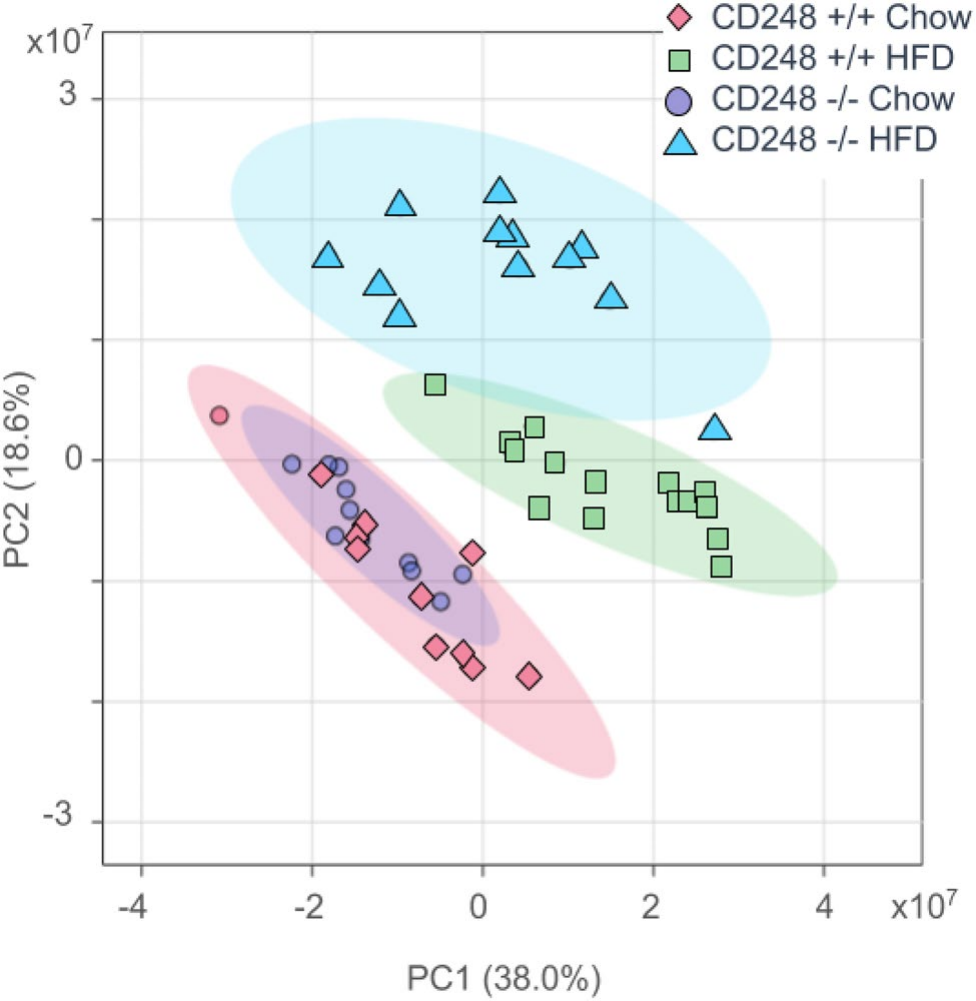
PCA analysis scores plot generated using MetaboAnalyst software of 5596 ion signals from all samples, analysed in positive ion mode (data mean-centred). Comparison of PCA scores for all features extracted using HRAM UHPLC–MS in positive ESI between CD248^+/+^ (pink diamonds) and CD248^−/−^ (purple circles) on normal chow or CD248^+/+^ (green squares) and CD248^−/−^ (blue triangles) on HFD

Given that food intake was similar between the genotypes, but lipid storage in fat pads was reduced in the CD248^−/−^, we assessed the relative amounts of circulating lipids using an un-targeted HRAM UHPLC–MS method. QC samples of pooled serum were used to ensure stability of the method. Samples were analysed in a single batch, to exclude any batch effects, and the precision of the analytical data was ascertained, initially by the PCA plot, which exhibited tight clustering of the QC samples (Fig. S2). The serum lipid profiles of 23 week old CD248^−/−^ and CD248^+/+^ mice on chow diet appeared equivalent with no discrimination by genotype apparent by PCA. However, following 13 weeks of HFD feeding, multiple differences were detected between the lipid profiles of the two genotypes and the groups were readily separated by PCA (Fig. 3). Metabolic profiles between male and female mice was assessed through PCA (data not shown) and, as no differences were noted, both sexes were grouped in all subsequent analyses. Cage effects that might have resulted from the way in which the animals were housed (in sub-groups of 4–6 animals according to sex, genotype and diet as described in the experimental section) were also assessed with no differences observed between them (data not shown).

#### Lysophosphatidylcholines and phosphatidylcholines

**Fig. 4:**
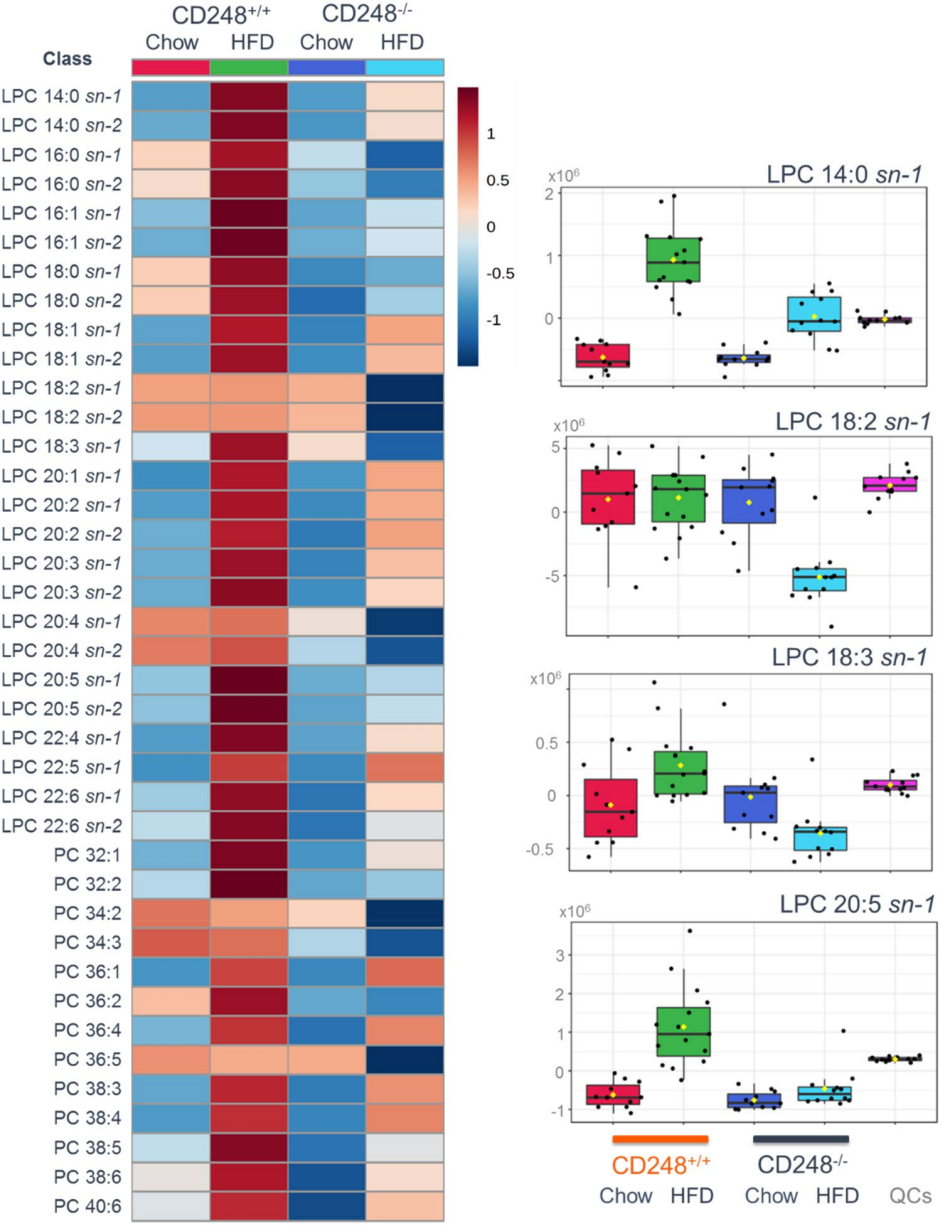
Heatmap generated using MetaboAnalyst highlighting the differential responses of LPC and PC species in the serum to high fat diet (data mean-centred). Peak area data scaled and plotted on a divergent scale. Relative expression is shown with high expression in red and low expression in dark blue. The boxplots inset to the right illustrate the various different types of behaviour displayed by these lipidic species: Chow-fed CD248^+/+^ (red) and CD248^−/−^ (dark blue); HFD-fed CD248^+/+^ (green) and CD248^−/−^ (light blue). Quality control samples (QCs, magenta) on the boxplots demonstrate the degree of analytic variability for each LPC shown. Significance measured by ANOVA p < 0.05. For the boxplots shown: LPC 14:0 sn-1 p = 1.10 × 10^−14^; LPC 18:2 sn-1 p = 5.43 × 10^−07^; LPC 18:3 sn-1 p = 1.12 × 10^−04^; LPC 20:5 sn-1 p = 1.74 × 10^−09^. All p-values and results from Fisher’s post hoc tests are shown in Supplementary Table 1. LPC were identified as the sn-1 form based on their elution order compared to the sn-2 isomers (sn-2 first with sn-1 second)

UHPLC–MS revealed that the CD248^−/−^ genotype is characterised by statistically significant changes in serum lysophosphatidylcholine (LPC) and phosphatidylcholine (PC) composition compared to the controls. In WT mice, the relative amounts of LPC and PC lipids were significantly increased following maintenance on a HFD compared to the chow diet, as shown in the heatmap (Fig. 4). However, this heatmap also clearly demonstrates that CD248 knockout mice were protected from increased quantities of circulating lipids, despite ingestion of similar quantities of fat in their diet (Fig. 2). Within this broad phenotype there were some nuanced changes in affected lipids, examples of which are highlighted in the charts in Fig. 4. Such examples include, LPC 18:2 sn-1 which was significantly reduced in relative amounts in CD248^−/−^ mice on the HFD compared to WT animals, whilst it appeared to be unaffected by a HFD. Similarly, LPC 18:3 sn-1 was reduced in CD248^−/−^ mice on HFD whilst it was raised in WT mice under the same conditions. Broadly, of the 26 LPCs shown to increase in relative quantities in WT mice in response to HFD, a majority showed a decrease in the CD248^−/−^ animals. Whilst the remainder of the LPCs were increased in relative amount in the serum of CD248^−/−^ mice on the HFD compared to their chow-fed comparators, this was to a lesser extent than had occurred in the WT controls. Closer examination of these data show that the effect of HFD on lipids in the circulation, for both CD248^−/−^ and CD248^+/+^ animals, comprised a mixture of strain-dependent responses from a reduction, little change or an increase in relative amounts.

**Fig. 5:**
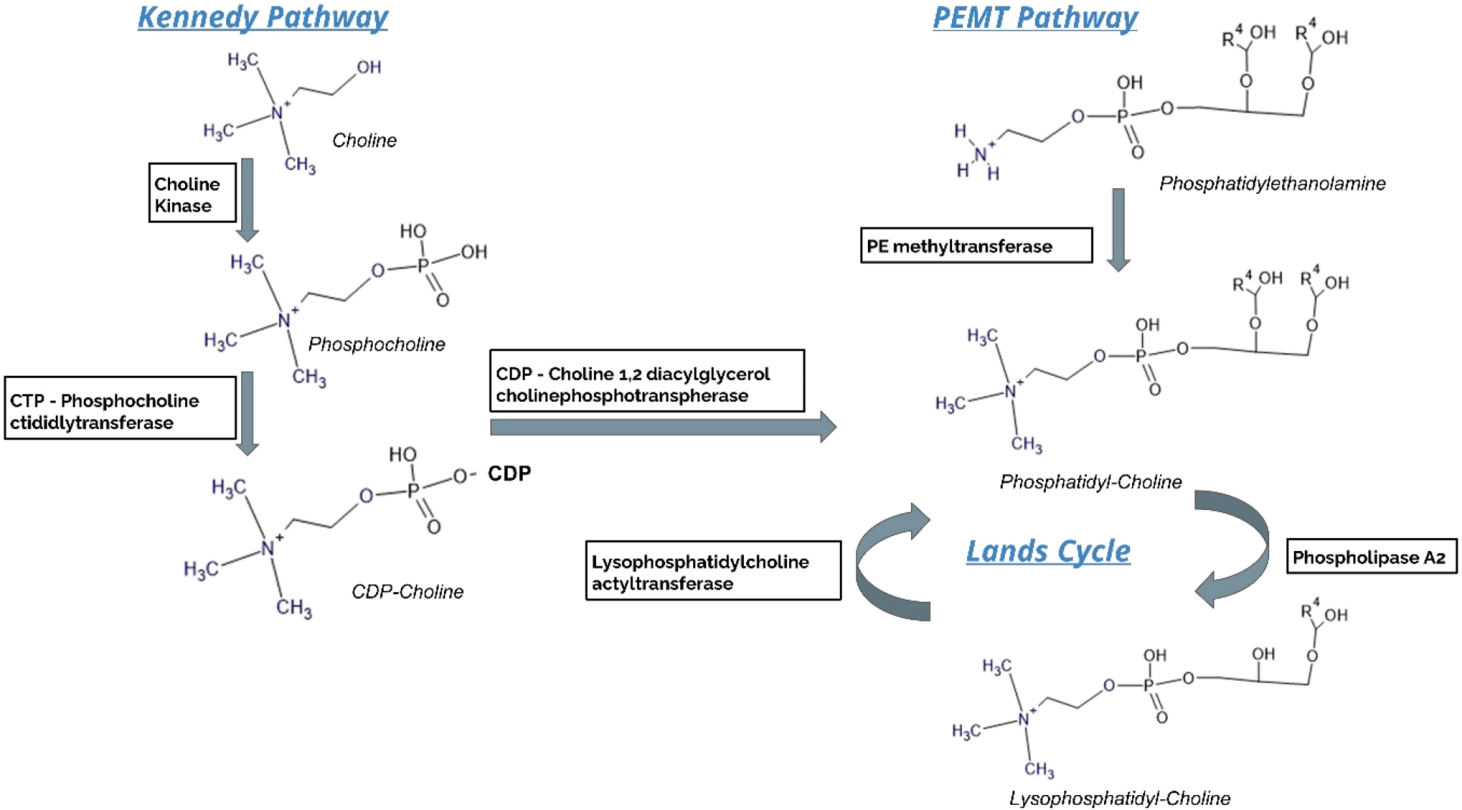
Outline of the Kennedy pathway, PEMT pathway and the Lands cycle. The substrates and enzymes involved are highlighted. Alterations in the uptake of substrates and/or activity or gene expression of enzymes could explain the observed phenotype and requires further investigation

As is well established, PCs are the most abundant glycerophospholipids in mammalian cell membranes and, at their most basic level, act to balance the proportion of bilayer lipids that determine membrane intrinsic curvature (De Kroon 2007). PC can be synthesised via two pathways; either starting from choline via the Kennedy pathway (Kennedy and Weiss 1956), which accounts for the majority of PC synthesis (70%), or from phosphatidylethanolamine (PE) via the phosphatidylethanolamine *N*-methyltransferase (PEMT) pathway (Sundler 1975) which in turn is responsible for around 30% of PC biosynthesis (Vance 2014). These two pathways feed into the Lands cycle (1958) whereby, glycerophospholipids are acetylated and deacetylated. For instance, LPC are synthesised from PC by circulating phospholipase A_2_ (PLA_2_) and in the reverse process, lysophosphatidylcholine acetyltransferase (LPCAT) utilises acetyl-CoA to convert LPC back to PC (Fig. 5) (Shindou and Shimizu 2009). Clearly, given the differential response seen between the two mouse strains, the CD248 gene has some influence on regulating aspects of these important biosynthetic pathways.

#### Cholesterol, choline and carnitine

**Fig. 6:**
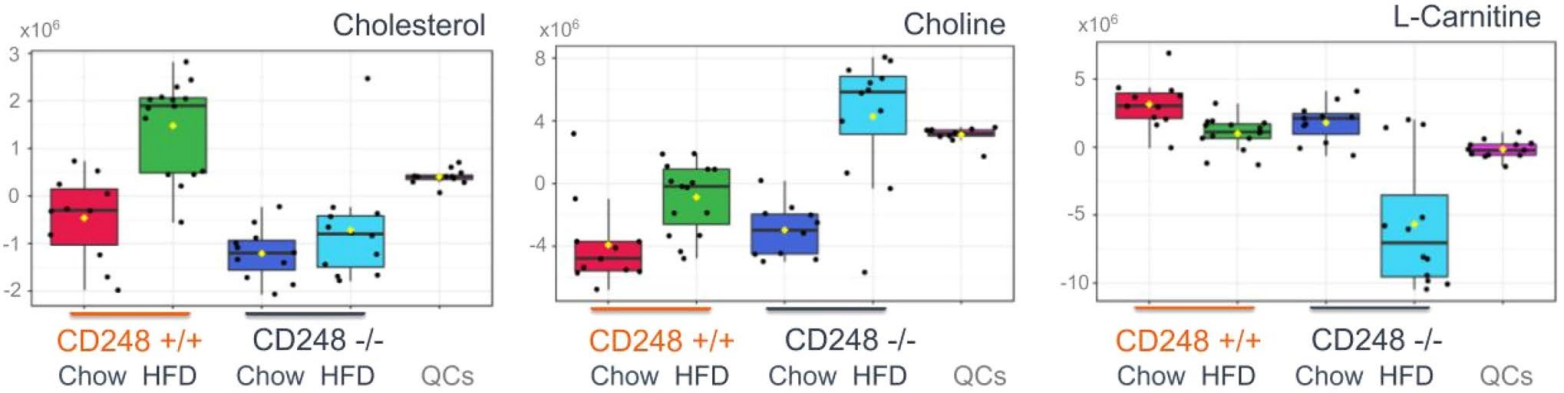
Box plots generated using MetaboAnalyst showing changes in cholesterol, choline and carnitine that significantly differed in response (peak area) to the HFD fat diet between the CD248^−/−^ and control mice (data mean-centred). Chow-fed CD248^+/+^ (red) and CD248^−/−^ (dark blue); HFD-fed CD248^+/+^ (green) and CD248^−/−^ (light blue). Quality control samples (QCs, magenta) on the boxplots demonstrate the degree of analytic variability for each LPC shown. Significance measured by ANOVA p < 0.05. Cholesterol p = 6.76 × 10^− 09^; choline p = 2.98 × 10^−08^; l-carnitine p = 9.54 × 10^−10^. Results from Fisher’s post hoc tests are shown in Supplementary Table 1

A significant difference was also observed in the relative amounts of serum cholesterol, choline and carnitine for mice fed the high fat diet: CD248^−/−^ mice did not show the same marked increase in the relative amounts of serum cholesterol as the CD248^+/+^ controls on HFD, whilst demonstrating a statistically significant increase in serum choline and a marked reduction in serum carnitine compared to controls (Fig. 6).

Reductions in circulating carnitine are supportive of alterations in fatty acid oxidation as it is required for the transfer of long-chain fatty acids to mitochondria for subsequent β-oxidation (Yano et al. 2010). The increase in choline appears to be correlated with the observed changes in PCs and LPCs (Fig. 4). In addition, the lack of elevation in serum cholesterol in CD248^−/−^ HFD-fed mice, in sharp contrast to the response of the WT animals, is consistent with the reduced propensity for CD248^−/−^ mice to suffer from atherosclerosis under these conditions (Hasanov et al. 2017).

Furthermore, changes were detected in other classes of metabolites including some fatty acid and fatty acid methyl ester metabolites (Supplementary Fig. S4), lysophosphatidylethanolamines (LPE) and phosphatidylethanolamines (PE) (Supplementary Fig. S5) and cholic acid, glycerophosphocholine, valine/betaine (Supplementary Fig. S6).

### CD248 genetic status predominantly affects the circulating composition and amounts of LPC and PC

Clearly this work highlights significant differences in glycerophospholipid handling between the two strains of mice and clearly points towards altered regulation of lipid metabolism/biosynthesis in CD248^−/−^ mice compared to their CD248^+/+^ equivalents. Although the exact function of CD248 in this process is not easily interpreted, due to the interconnectedness of the pathways involved, the results clearly show the most affected lipid class to be the PCs and LPCs.

In addition to their role as membrane components, PCs are known to regulate insulin receptors by activation of phosphatidylcholine transfer protein and subsequent downstream interactions with mTORC1 and diacylglycerol kinase δ (Ersoy et al. 2013; Sakai et al. 2014) and may help to regulate blood glucose concentrations. Indeed, CD248 has recently been linked to control of insulin sensitivity but its mechanism of action has yet to be elucidated (Petrus et al. 2019). LPCs are known to interconvert with PCs and signal via PPARs (Singh 2017) and, given that the PPARs are regulators of fatty acid oxidation and adipocyte differentiation, the differences in lipid accumulation in the peri-renal fat pad between control and CD248^+/+^ could be one result of such an interaction. It is possible to conjecture that CD248 has an interaction with lipids such as LPC 18:3 sn-1, or other LPCs, on the level of a signalling pathway. Whilst CD248 appears to be required for efficient platelet derived growth factor (PDGF) BB signalling via the PDGF receptor β (Tomkowicz et al. 2010) the mechanism is as yet unexplained. It may be that this interaction is mediated by PCs or LPCs in a similar manner to that seen with the insulin receptors. Given that the accumulation of LPCs is associated with atherosclerotic plaque (Kritikou et al. 2016; Yoshida et al. 2003) the minimal increases in the relative amounts of these lipids in the circulation of the CD248^−/−^ mice is consistent with previously reported protection of these animals from plaque formation (Hasanov et al. 2017).

Alterations in the Lands cycle (1958) may also be the cause of the changes seen in several fatty acids (FAs) and fatty acid methyl esters (FAMES) (Fig. S3) as these are a product of PLA2 converting PC to LPC. The modest increases in some lysophosphatidylethanolamine (LPE) and phosphatidylethanolamine (PE) noted for CD248^−/−^ on a HFD compared to wildtype (Fig. S5) could suggest a decrease in activity of the PEMT pathway which normally converts PE to PC.

## Conclusions

CD248 (Endosialin/Tumour Endothelial Marker 1) has previously been identified as a protein upregulated on mesenchymal cells in a number of human diseases (Wilhelm et al. 2016; Park et al. 2020), and recently in obesity (Petrus et al. 2019). Untargeted metabolic phenotyping of serum from the CD248^−/−^ and C57Bl/6^+/+^ (WT) controls revealed very different metabolic responses to a high fat diet. These differences, particularly in lipid handling by the CD248^−/−^ knockout mice, provide clues to the mechanisms by which protection from atherosclerosis (Hasanov et al. 2017) is achieved.

We conjecture that these differences in the serum metabolic profiles and fat deposition in visceral fat pads between control and CD248 knockout animals, particularly with respect to the LPCs and PCs, result from differential regulation of biosynthesis in systems such as the Kennedy (Kennedy and Weiss 1956), PEMT pathways (Sundler 1975) or Lands cycle (1958). The results detailed here provide a good case for examining the activity and localisation of the LPCAT isoforms, PLA2, choline kinase and choline-phosphate cytidylyltransferases at the gene expression and enzymatic activity, which might provide a mechanistic explanation for the reduced amounts of cholesterol, LPC, PC, l-carnitine and increased choline in serum from CD248^−/−^ mice reared on a HFD.

## Supplementary information

Below is the link to the electronic supplementary material.

(DOCX 1413 kb)

(XLSX 62 kb)

## Data Availability

All relevant metabolomics data are provided in the Supplementary Information.
