# Supplementary material for: Metabolic consequences for mice lacking Endosialin: LC–MS/MS-based metabolic phenotyping of serum from C56Bl/6J Control and CD248 knock‐out mice
Source: Metabolomics. 2021 Jan 18;17(2):14. doi: 10.1007/s11306-020-01764-1 (PMC7813710; doi:10.1007/s11306-020-01764-1)
Supplement: Supplementary file 1 — (DOCX 1413 kb) [file 11306_2020_1764_MOESM1_ESM.docx]

**Supplementary Figures**

**
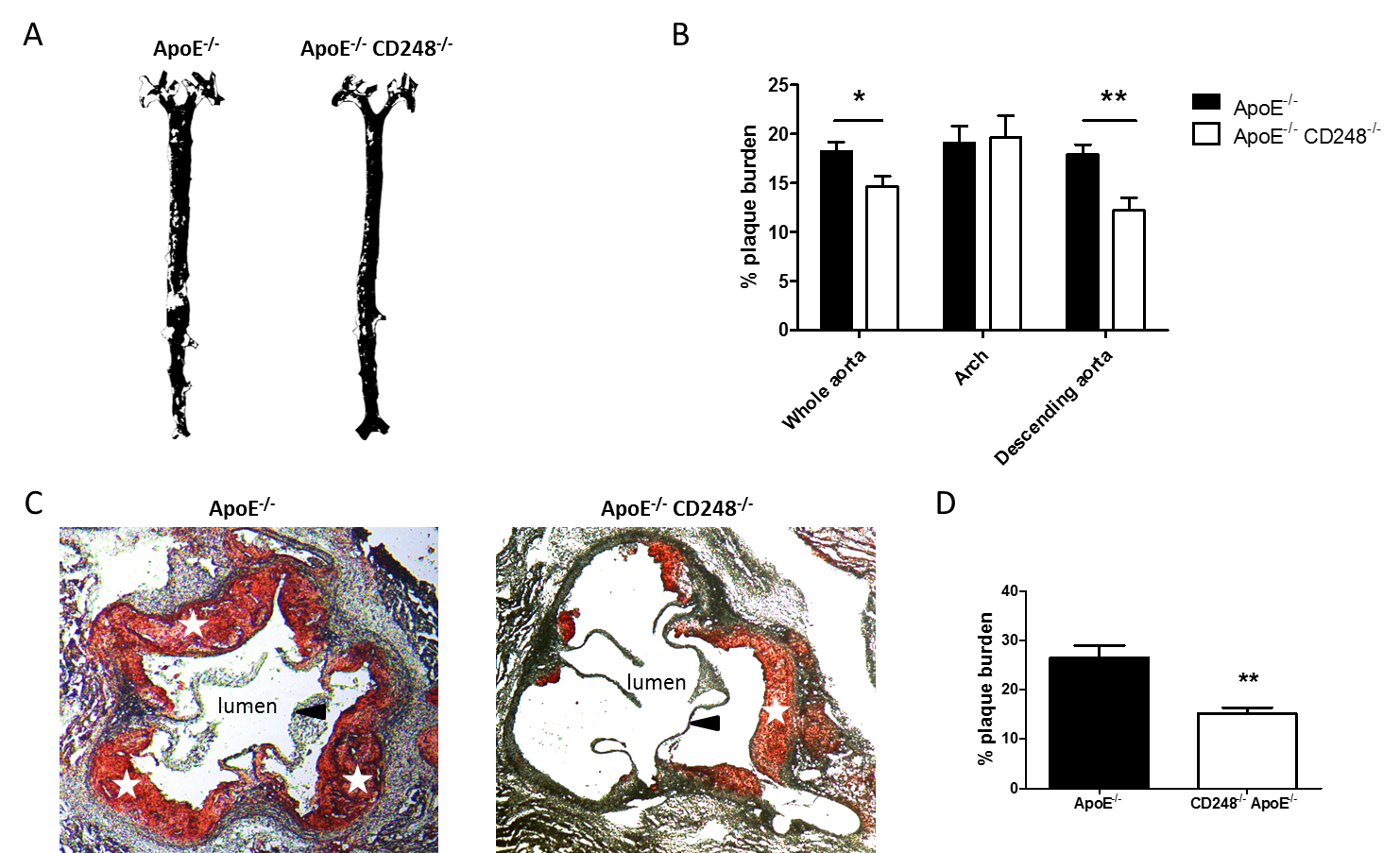
**

**Figure S1.** Plaque burden assessment in aortas of ApoE^-/-^ and CD248 ApoE^-/-^ mice.

**A.** Representative (false colour) images of oil-red O stained aorta from ApoE^-/-^ and ApoE^-/-^ CD248^-/-^ mice. Plaques are shown in white. **B.** Percentage plaque burden was calculated for the whole aorta, the arch and the descending aorta. **C.** Representative images of oil red o-stained cross sections of aortic root in ApoE^-/-^ mice and CD248^-/-^ ApoE^-/-^ mice. **D.** Quantification of plaque burden using ImageJ analysis. Data are expressed as mean ± SEM, * P<0.05, **P<0.01.


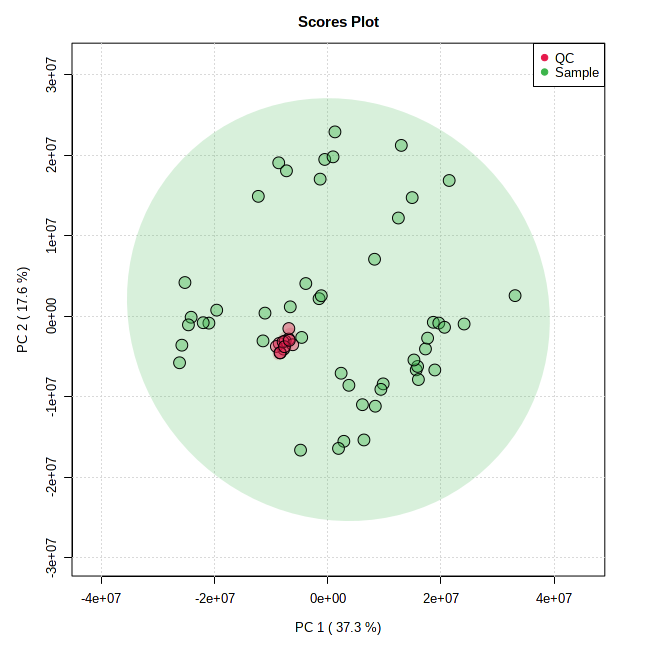


**Figure S2**. Distribution of serum sample from CD248^+/+^ and ^-/-^ mice (green) and pooled QCs (red) showing good clustering of the latter, with no time-related trends for the QC data.


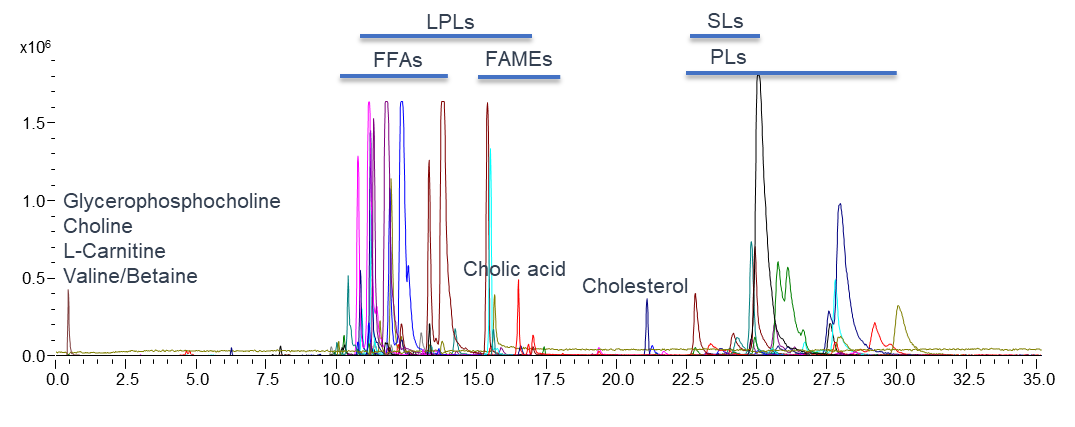


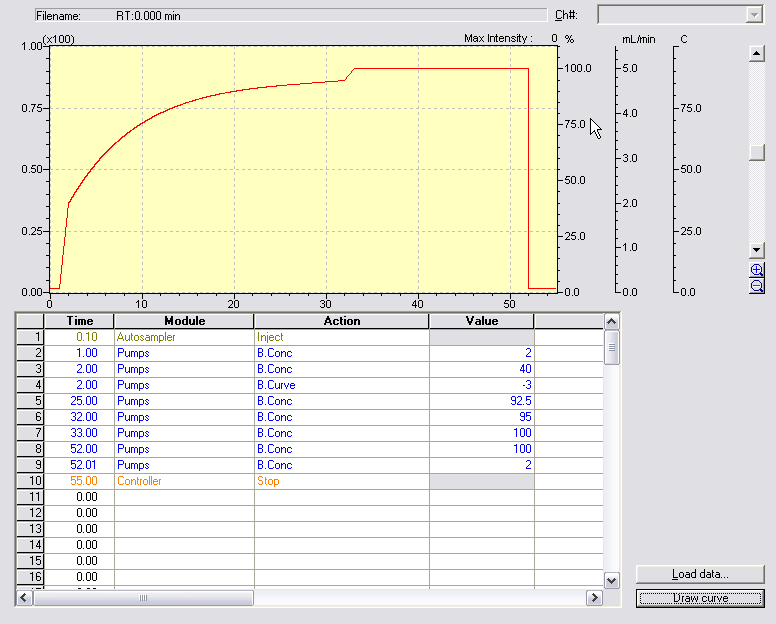


**Figure S3.** Upper figure shows the precursor HRAM mass chromatograms of all the annotated components detected in the pooled QC serum extract. LPLs (lysophospholipids), SLs (sphingolipids), FFAs (free fatty acids) and FAMEs (fatty acid methyl esters), in addition to cholesterol, L-carnitine, choline and pantothenic acid. Lower figure shows the non-linear concave gradient profile (setting -3 in LabSolutions) used in the LC separation.


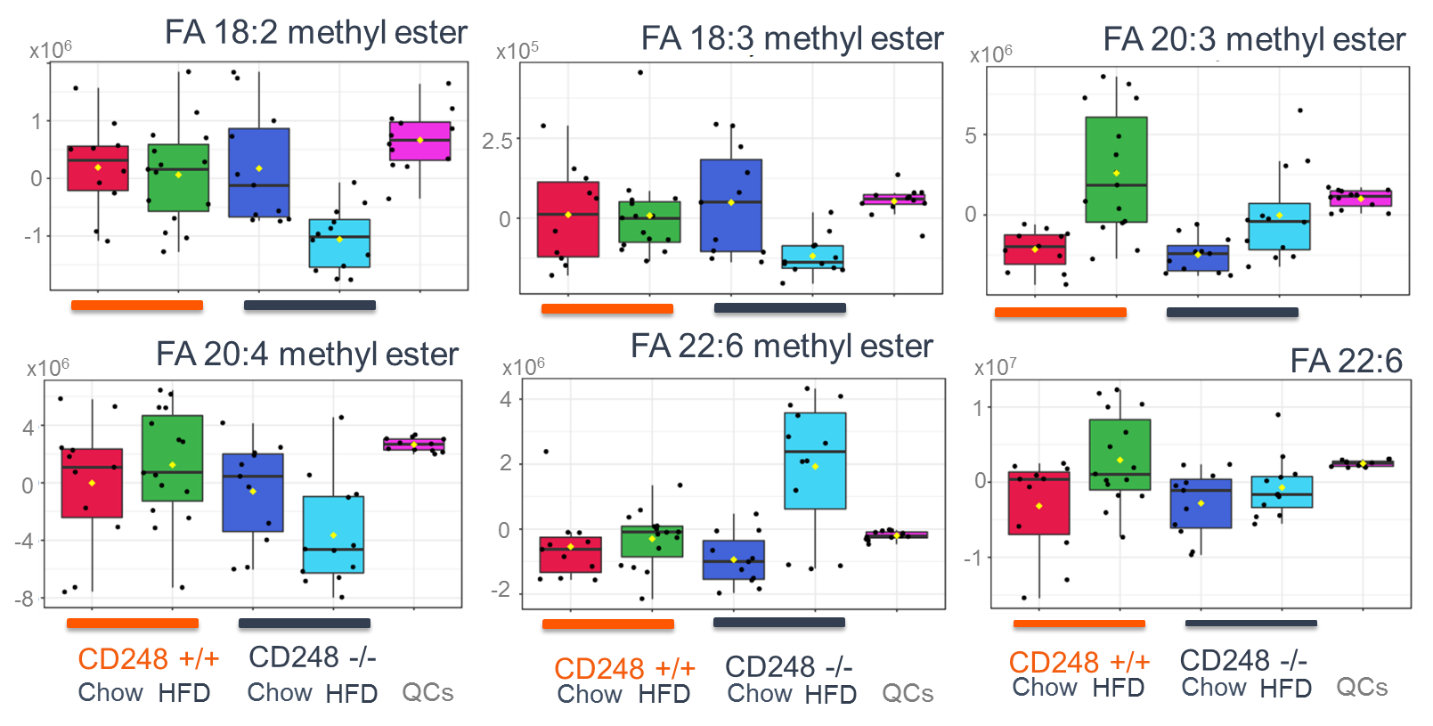


**Figure S4.** Box and whisker plots generated using Metaboanalyst presenting fatty acid and fatty acid methyl ester metabolites that significantly differed in response (peak area) to the HFD fat diet between the CD248^-/-^ and CD248^+/+^ control mice (data mean-centred). Significance measured by ANOVA p<0.05: FA 18:2 methyl ester p=7.66×10^-04^; FA 18:3 methyl ester p=0.02; FA 20:3 methyl ester p=4.03×10^-05^; FA 20:4 methyl ester p=0.02; FA 22:6 p=9.45×10^-06^; FA 22:6 methyl ester p=0.02. Results from Fisher’s post hoc tests are shown in supplementary table 1.


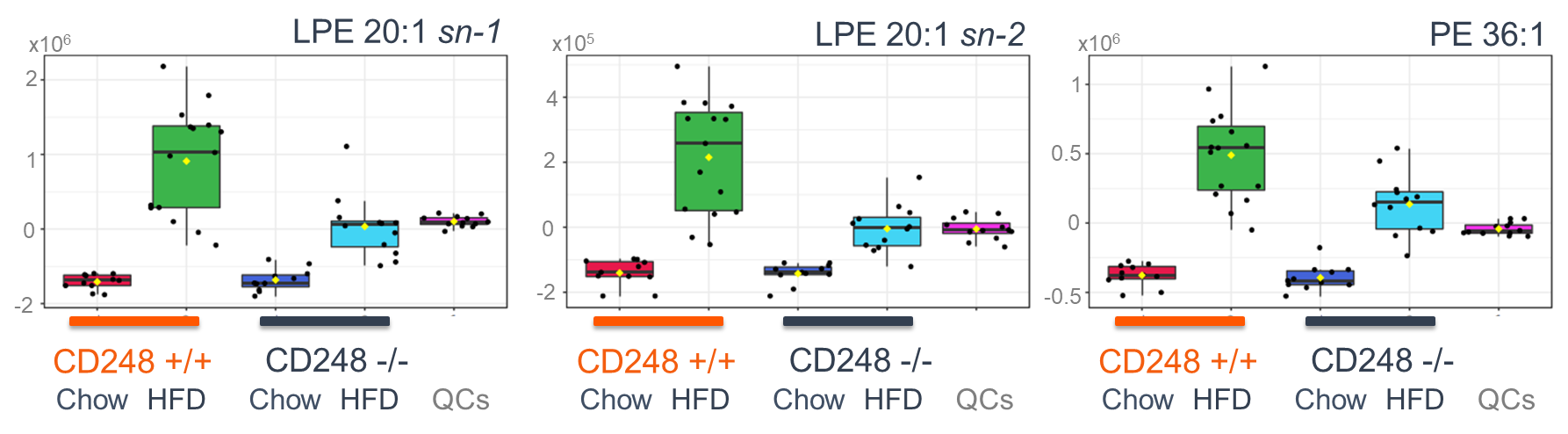


**Figure S5.** Box and whisker plots generated using Metaboanalyst showing changes to lysophosphatidylethanolamine (LPE) and phosphatidylethanolamine (PE) that that that significantly differed in response (peak area) to the HFD fat diet between the CD248^-/-^ and CD248^+/+^ control mice (data mean-centred). Significance measured by ANCOVA p<0.005: LPE 20:1 sn-1 p=7.85×10^-12^; LPE 20:1 sn-2 p=2.15×10^-11^; PE 36:1 p=6.38×10^-14^. Results from Fisher’s post hoc tests are shown in supplementary table 1.


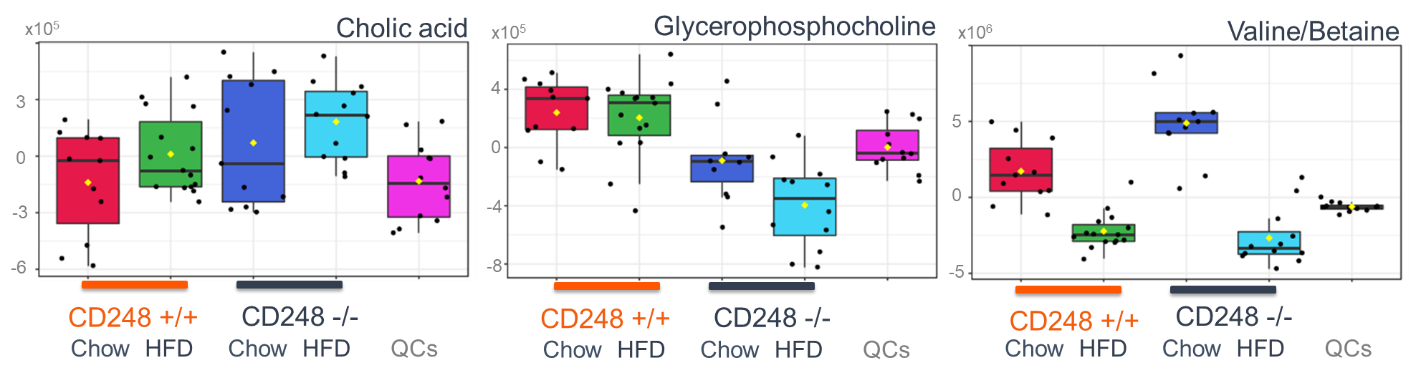


**Figure S6.** Box and whisker plots generated using Metaboanalyst highlighting cholic acid, glycerophosphocholine and a peak corresponding to either valine or betaine metabolites that significantly differed in response (peak area) to the HFD fat diet between the CD248^-/-^ and CD248^+/+^ control mice (data mean-centred). Significance measured by ANOVA p<0.05: Cholic acid p=0.04; Glycerophosphocholine p=8.66×10^-07^; Valine/Betaine p=4.33×10^-13^. Results from Fisher’s post hoc tests are shown in supplementary table 1.
